# Loss of miR-145-5p Causes Ceruloplasmin Interference with PHD-Iron Axis and HIF-2α Stabilization in Lung Adenocarcinoma-Mediated Angiogenesis

**DOI:** 10.3390/ijms21145081

**Published:** 2020-07-18

**Authors:** Ying-Ming Tsai, Kuan-Li Wu, Yung-Yun Chang, Wei-An Chang, Yung-Chi Huang, Shu-Fang Jian, Pei-Hsun Tsai, Yi-Shiuan Lin, Inn-Wen Chong, Jen-Yu Hung, Ya-Ling Hsu

**Affiliations:** 1 Graduate Institute of Medicine, College of Medicine, Kaohsiung Medical University, Kaohsiung 807, Taiwan; tsaiyingming@gmail.com (Y.-M.T.); 980448kmuh@gmail.com (K.-L.W.); beryl1992@gmail.com (Y.-C.H.); chienfang1216@gmail.com (S.-F.J.); kanginbobo@hotmail.com (P.-H.T.); ysirenelin@gmail.com (Y.-S.L.); chong@kmu.edu.tw (I.-W.C.); yainghsu@kmu.edu.tw (Y.-L.H.); 2 Department of Internal Medicine, School of Medicine, College of Medicine, Kaohsiung Medical University, Kaohsiung 807, Taiwan; cyy807@gmail.com (Y.-Y.C.); 960215kmuh@gmail.com (W.-A.C.); 3 Division of Pulmonary and Critical Care Medicine, Department of Internal Medicine, Kaohsiung Medical University Hospital, Kaohsiung Medical University, Kaohsiung 807, Taiwan; 4 Department of Respiratory Care, College of Medicine, Kaohsiung Medical University, Kaohsiung 807, Taiwan; 5 Drug Development and Value Creation Research Center, Kaohsiung Medical University, Kaohsiung 807, Taiwan; 6 Department of Internal Medicine, Kaohsiung Municipal Ta-Tung Hospital, Kaohsiung Medical University, Kaohsiung 807, Taiwan

**Keywords:** angiogenesis, ceruloplasmin, HIF-2α, lung cancer, miR-145-5p

## Abstract

For decades, lung cancer has been the leading cause of cancer-related death worldwide. Hypoxia-inducible factors (HIFs) play critical roles in mediating lung cancer development and metastasis. The present study aims to clarify how HIF’s over-activation affects lung cancer angiogenesis not only in a normoxic condition, but also a hypoxic niche. Our study shows that human lung cancer exhibits elevated levels of ceruloplasmin (CP), which has a negative impact on the prognosis of patients. CP affects the cellular Fe^2+^ level, which inactivates prolyl hydroxylase (PHD) 1 and 2, resulting in HIF-2α enhancement. Increased HIF-2α leads to vascular endothelial growth factor-A (VEGF-A) secretion and angiogenesis. The expression of CP is under the epigenetic control of miR-145-5p. Restoration of miR-145-5p by miRNA mimics transfection decreases CP expression, increases Fe^2+^ and PHD1/2 levels and HIF hydroxylation while reduced HIF-2α levels resulting in the inhibition of tumor angiogenesis. In contrast, inhibition of miR-145-5p by miRNA inhibitors increases the expression of CP and VEGF-A in lung cancer cells. Significantly, miR-145-5p expression is lost in the tumor samples of lung cancer patients, and low miR-145-5p expression is strongly correlated with a shorter overall survival time. In conclusion, the current study reveals the clinical importance and prognostic value of miR-145-5p and CP. It identifies a unique mechanism of HIF-2α over-activation, which is mediated by iron imbalance of the iron-PHD coupling that modulates tumor angiogenesis.

## 1. Introduction

Lung cancer is well-known as the most prevalent cancer worldwide [1]. Non-small cell lung cancer (NSCLC) makes up 75–80% of all primary lung cancer cases [2]. For decades, lung cancer has been ranked first for cancer-associated mortality in Taiwan [3]. Lung cancer is the cause of 17% and 9% of all cancer cases in men and women respectively and represents 19% of all cancer-related deaths worldwide [4]. The advent of novel therapeutic approaches such as tyrosine kinase inhibitors, immune checkpoint inhibitors might benefit patients of lung cancer [5]. Regrettably, lung cancer has a particularly low 5-year survival rate (ranging from 6 to 18%) compared with other cancers, such as breast (89%), prostate (99%) and colon carcinoma (65%) [6]. Improved understanding of the underlying mechanisms of lung cancer development is a first priority to help prolong the survival time of patients and could even help develop a cure.

Hypoxia-inducible factors (HIFs) were originally developed in simple multicellular animals to control cellular glycolysis or oxidative phosphorylation, which was dependent on O_2_ levels. HIFs target genes involving energy metabolism, cellular proliferation, erythropoiesis/iron metabolism and vascular development/remodeling [7,8]. HIF isoforms are characterized by different heterodimers that are composed of a constitutive HIF-1β and an α subunit (HIF-1α subunit for HIF-1α and HIF-2α subunit for HIF-2α). Both subunits contain basic helix-loop-helix domains, and mediate dimerization and DNA binding [9]. The stability of HIFs is primarily regulated by post-transcriptional modifications and proteasome degradation. Both HIF-1α and HIF-2α play a major role in mediating the tumorigenesis and progression of lung cancer [10,11,12]. In addition, HIF-2α has a distinct biological role from HIF-1α other than tumor formation mechanisms but also induces vascular endothelial pro-angiogenic factor expression [12]. Prolyl hydroxylase domain proteins (PHDs) are members of a large family of non-heme iron-dependent dioxygenases, which regulate HIF family proteins via hydroxylation in a normoxic condition [13]. The three main PHD isoforms, PHD1, PHD2 and PHD3 (also termed EGLN2, EGLN1 and EGLN3, respectively) have overlapping but unique tissue expression patterns. PHD2 is present in most tissues; PHD1 is present mainly in the testes but also in brain, kidneys, heart and liver; while PHD3 has highest expression in the heart [14,15]. The enzymatic activity of PHDs depends on ferrous iron (Fe^2+^) as the activating metal. Changes in iron levels regulate oncogenic signaling transductions such as HIFs, by changing the activation of PHDs in cancer. Therefore, iron plays a key role in the regulation of microenvironments and cancer metastasis.

Ceruloplasmin (CP) mediates several physiological functions such as copper transport, ferroxidase activity, superoxide dismutase activity and amine oxidase activity [16]. CP regulates the efflux of ferrous iron (Fe^2+^) by releasing iron from export protein ferroportin via oxidation Fe^2+^ into ferric iron (Fe^3+^) [17,18]. Individuals with aceruloplasminemia, the absence of circulating serum CP, accumulate iron in vital organs such as the liver and basal ganglia, typically suffer from tissue injury and necrosis in their fourth to fifth decades of life [19]. The aim of this study is to provide the relationship among HIFs, CP, iron and lung cancer. Therefore, we propose that elevated expression of CP modulates Fe^2+^ levels and affects the HIFs degradation, the present study is performed to determine the role of CP in mediating lung cancer angiogenesis, as well as its underlying regulatory mechanisms, and these results might provide the further applications in lung cancer.

## 2. Results

### 2.1. High Levels of CP Contribute to Carcinogenesis and Cancer Progression

**Figure 1:**
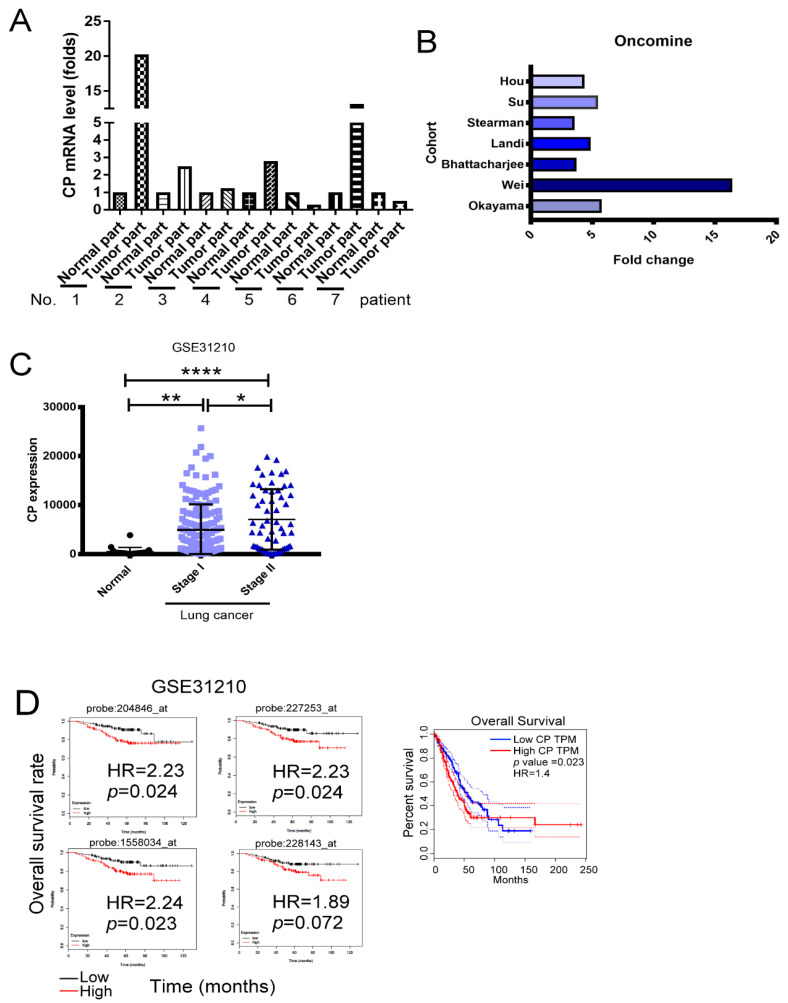
Ceruloplasmin (CP) protein is upregulated in lung cancer and correlated with poor overall survival rate. (**A**) Upregulated CP in the tumor of 7 lung cancer patients. (**B**) The elevated levels of CP in lung cancer patients are found from seven independent microarrays retrieved from the Oncomine^®^ database. (**C**) The level of CP in lung cancer patient (GSE31210, 20 normal, 168 stage I and 57 stage II lung cancer tissue). (**D**) The relationship of CP protein with the clinical outcome of lung cancer patients. * *p*  <  0.05, ** *p*  <  0.01, **** *p*  <  0.0005.

CP has been linked to carcinogenesis and cancer progression [20]; however, the underlying molecular mechanisms of CP remain unknown. To determine whether CP is associated with lung cancer, the expression of CP in seven pairs of normal lung and adenocarcinoma lung tissue samples was assessed. In these seven pairs of human specimens, higher levels of CP mRNA were found in 4 out of the 7 tumor samples compared with the normal samples, as determined by RNA-seq (Figure 1A). In addition, the 7 datasets identified from the Oncomine^®^ database also showed that the levels of CP expression were increased in lung cancer tissue samples compared with normal tissue samples (Figure 1B).

To further validate whether the CP level was positively associated with lung cancer progression, the GSE31210 dataset from the Gene Expression Omnibus (GEO) was analyzed. This dataset was a lung cancer cohort, containing normal individuals, those with *epidermal growth factor receptor (EGFR)* mutations, *anaplastic lymphoma kinase (ALK)* mutations, *RAS* mutations and wild type lung adenocarcinoma at different stages. The results revealed that the expression of *CP* was significantly higher in tumor tissues compared with adjacent non-tumor tissues. Significantly higher *CP* levels were observed in the tumors of stage II patients compared with the tumors of stage I patients (Figure 1C). Together, these findings indicate that *CP* expression is higher in advanced-stage lung adenocarcinoma.

Overall survival analyses of Kaplan Meier (KM)-plotter (left) and Gene Expression Profiling Interactive Analysis (GEPIA) (right) suggested that individuals with higher levels of CP expression had significantly shorter survival time compared with lung cancer patients with lower CP levels statistically (Figure 1D). In conclusion, these results indicate that CP expression is elevated in lung cancer patients and higher levels of CP expression are associated with a shorter survival time.

### 2.2. CP Affects HIF-2α Levels in Lung Cancer Cells via Iron/PHDs

**Figure 2:**
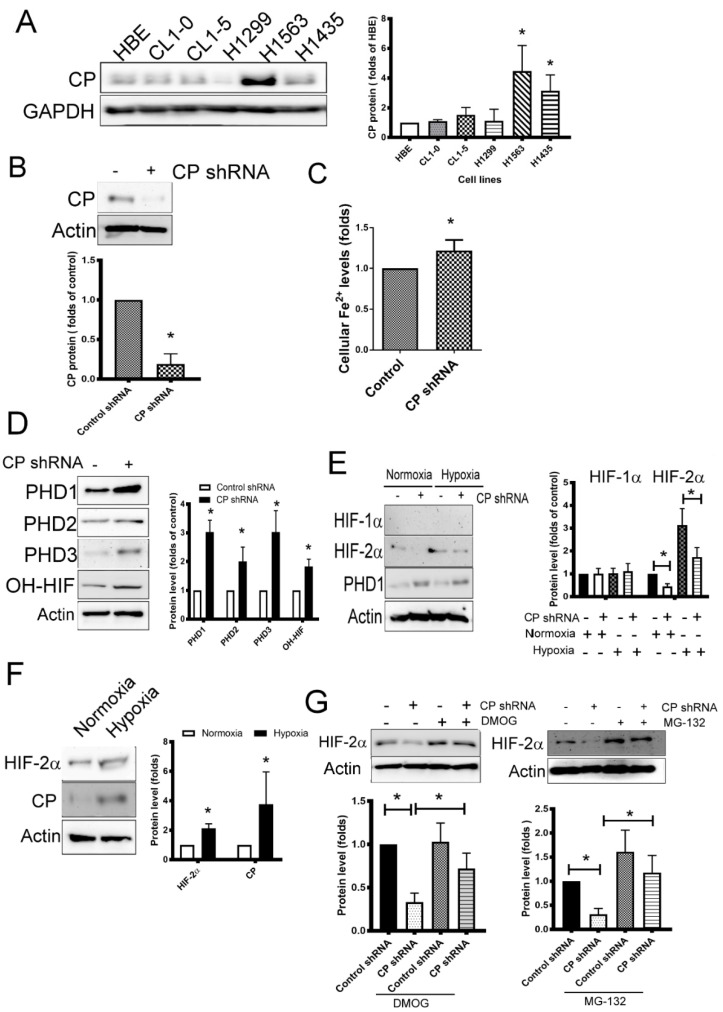
CP increases the stabilization of hypoxia-inducible factor (HIF)-2α. (**A**) The expression of CP in human bronchial epithelia (HBE) and various lung cancer cell lines. (**B**) The level of CP protein in CP-knockdown H1563 cells. (**C**) The cytosolic Fe^2+^ levels of CP-knockdown H1563 cells. (**D**) The level of prolyl hydroxylase domains (PHDs) and *hydroxylated HIF* (OH-HIF). (**E**) The levels of HIFs and PHD1 in CP-knockdown H1563 cells under either a normoxic or hypoxic condition. (**F**) Hypoxia increased CP and HIF-2α protein expression in H1563 cells. (**G**) CP increased HIF-2α level in a PHD-dependent and proteasome-dependent manners. H1563 cells were transfected with control or CP shRNA plasmid, and stable clones were established by puromycin selection. H1563 cells were treated with DMOG (75 μM) and MG-132 (10 μM) for 24 h. The levels of specific protein were detected by Immunoblot. Result is representative of at least three independent experiments. * *p*  <  0.05.

HIFs are transcription factors, which have been implicated as the major drivers of cancer progression and could be activated with various factors such as pH, nutrients and growth factors other than hypoxia [21]. The regulation of HIF degradation is dependent on iron, and CP stimulates iron efflux from the cell by releasing iron from iron export protein ferroportin via oxidation of ferrous (Fe^2+^) into ferric (Fe^3+^) [17,18]. Human normal bronchial epithelia (HBE) and lung cancer cell lines (CL1-0, CL1-5, H1299, H1563 and H1435) were used to test the expression of CP protein. H1563 expressed highest levels of two spliced forms CP (secreted CP, sCP) and membrane-bound glycosylphosphatidylinositol-anchored (GPI-Cp) compared with HBE (data not shown). Consistently, H1563 cancer cells also expressed elevated levels of CP (Figure 2A), we thus selected this lung cancer cell line to investigate the role of CP by a loss-of-function mode. Knockdown of CP expression via transfection with short hairpin (sh) RNA plasmid decreased CP protein, and its two spliced variants (sCP and GPI-CP) in H1563 cells (Figure 2B, Figure S1A–C). The level of cytosolic Fe^2+^ increased after CP knockdown in H1563 cells (Figure 2C). PHD activity is dependent on oxygen and Fe^2+^ [22]. Decreased CP expression led to elevated PHD1, PHD2 and PHD3 levels; this was consistent with an increase in HIF hydroxylation (Figure 2D). In addition, our results showed that CP could regulate HIF-2α stabilization in by changing PHD1 levels and hydroxylation function under either a normoxic or hypoxic condition (Figure 2E). Hypoxic stimulation increased the expression of CP and HIF-2α in H1563 cells (Figure 2F). Moreover, both a PHD inhibitor dimethyloxalylglycine (DMOG) and a proteasome inhibitor (MG-132) reversed the HIF-2α degradation after CP inhibition in H1563 cells (Figure 2G). These results show that knockdown of CP disrupted the efflux of Fe^2+^, which then activated PHDs, which in turn resulted in decreasing levels of HIF-2α in H1563 lung cancer cells.

### 2.3. CP Enhances Angiogenesis via VEGF-A

**Figure 3:**
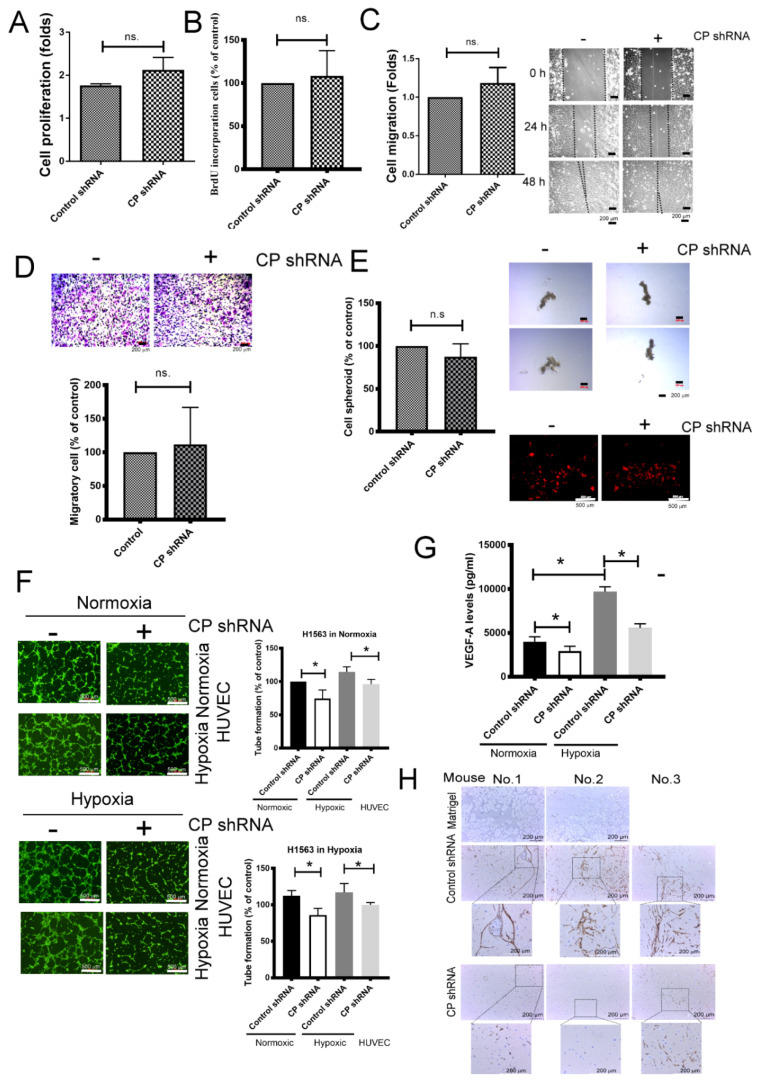
CP contributes to tumor angiogenesis. (**A**,**B**) The effect of CP in cell proliferation, as determined by Water Soluble Tetrazolium Salts (WST-1) and Bromodeoxyuridine (BrdU) incorporation. (**C**,**D**) The influence of CP inhibition on cell migration, as determined by wound healing and transwell system. (**E**) Inhibition of CP did not affect anchorage-independent cell growth of H1563 cells. (**F**) Decreased CP reduced tube formation in either a normoxic or hypoxic condition. (**G**) Inhibition of CP reduced vascular endothelial growth factor-A (VEGF-A) production. (**H**) Knockdown CP reduced angiogenesis in a mouse model. The proliferation of H1563 and CP-knockdown H1563 were measured by WST-1 and BrdU incorporation after 72 h incubation. Cells were seeded in the top of transwell insert (8 μm), and complete culture medium was added into the bottom well as chemoattractant for 48 h. The migratory cells were stained by crystal violet. Cells were stained by PKH26, and cultured in Ultralow-attachment plates for 7 days. The conditioned media of H1563 and CP-knockdown H1563 cells were collected after 48 h incubation. Human umbilical vascular endothelial cells (HUVECs) were seeded into Matrigel-coated well containing various conditioned media (50%). The tube formation was visible by Calcein-AM dye. The level of VEGF-A was determined by Luminex Assays. H1563 and CP-knockdown H1563 cells were mixed with the high concentration of Matrigel and then subcutaneously injected into nude mice (Martrigel only *n* = 2, others *n* = 6). After 28 days, the Matrigel plugs were collected and dissected, then analyzed by immunohistochemical (IHC) staining using CD34 antibody. All results are representative of at least three independent experiments and each value is the mean ± SD of three determinations; * *p* <  0.05. ns, not significant.

HIF-2α has been previously shown to control various cancer behaviors; the following experiments were performed to determine the pathologic roles of CP in lung cancer. As shown in Figure 3A, the proliferation of H1563 cancer cells was not significantly different between the knockdown CP group and the control group after 72 h incubation, as determined by both Water Soluble Tetrazolium Salts (WST-1) and Bromodeoxyuridine (BrdU) incorporation (Figure 3A,B). In addition, there were no significant differences between the migratory abilities of the control and the CP knockdown group, as determined via wound-healing and transwell assays (Figure 3C,D). Similarly, tumor spheroid formation did not show an increase in characters, such as aggregation and increased number in the CP knockdown group compared with the control shRNA plasmid transfected H1563 cells. Cancer stem cell characterization via PKH26 staining did not reveal any significant differences between the two groups (Figure 3E). These results indicate that CP did not regulate specific cellular behaviors, such as proliferation, migration and tumor spheroid formation in H1563 cells.

With regard to tumor angiogenesis, tube formation of human umbilical vascular endothelial cells (HUVECs) stimulated by H1563 cancer cells was assessed. The result showed that conditioned media collected from CP knockdown H1563 cells in either a normoxic or hypoxic condition notably decreased the formation of tube structures in HUVECs, no matter endothelial cell exposed in normoxia or hypoxia (Figure 3F). The conditioned media from the CP knockdown group had significantly lower levels of vascular endothelial growth factor-A (VEGF-A) in either a normoxic or hypoxic condition compared with the control group, but other angiogenic factors such as interleukin (IL)-8, platelet-derived growth factor (PDGF)-AA, Ang-1 and angiogenin did not show any significant changes (Table 1 and Figure 3G). Furthermore, an in vivo Matrigel plug angiogenesis assay revealed that knockdown of CP in H1563 cells attenuated angiogenesis in mice using CD34 immunohistochemical staining (Figure 3H). These findings suggest that CP increases tumor angiogenesis via VEGF-A, but does not affect proliferation, migratory ability or tumor spheroid formation.

### 2.4. miR-145-5p Regulates CP Expression in Lung Cancer

**Figure 4:**
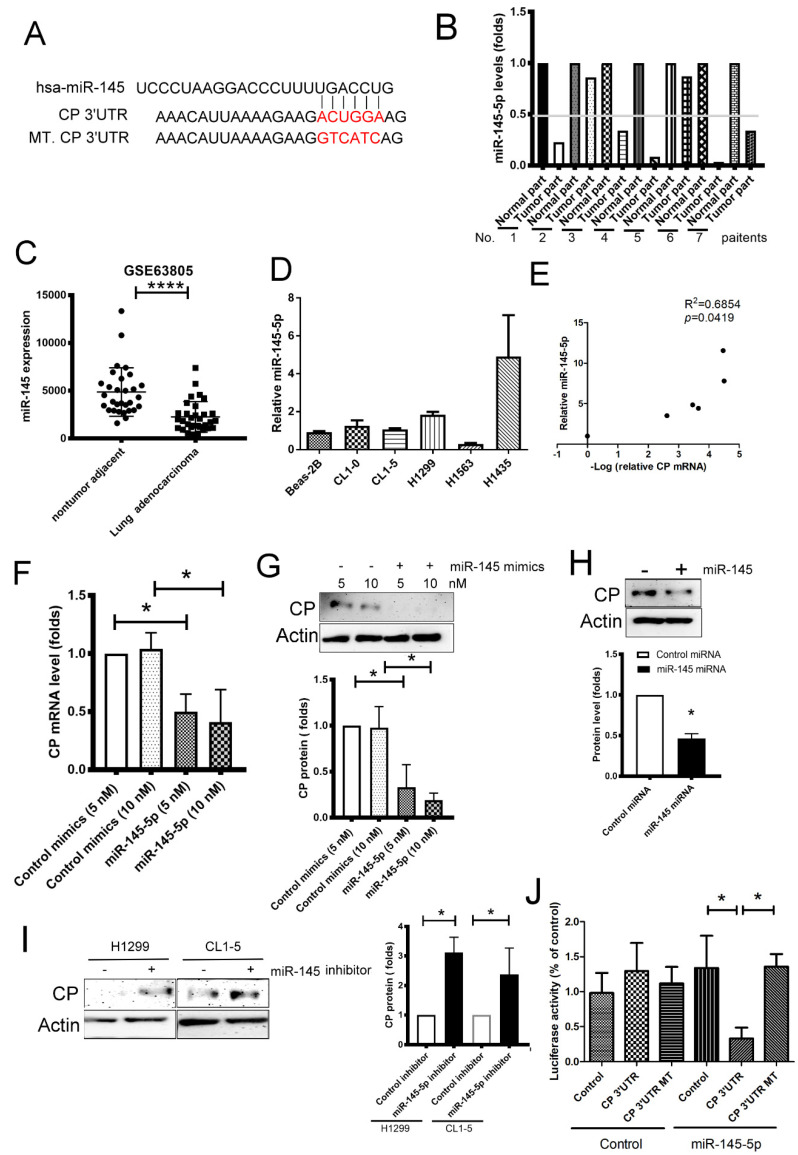
Loss of miR-145-5p contributes elevated CP protein in lung cancer. (**A**) The predicated binding site of miR-145-5p on the 3’untranslated region (UTR) of CP mRNA. (**B**) Downregulated miR-145-5p in tumor of 7 lung cancer patients. (**C**,**D**) The expression of miR-145-5p in lung cancer patients (GSE63805, 30 non-tumor adjacent tissue; 32 lung adenocarcinoma) and lung cancer cell lines. (**E**) The correlation of miR-145-5p and CP protein in Beas-2B cells and lung cancer cells lines. (**F**,**G**) miR-145-5p mimics decreased CP mRNA and protein expression in H1563 cells. (**H**) Overexpression of miR-145-5p decreased the expression of CP in human embryonic kidney (HEK)-293 cells. (**I**) miR-145-5p inhibitors increased CP protein expression in CL1-5 and H1299 lung cancer cell lines. (**J**) The luciferase activity of 3’UTR reporter analysis. The levels of miR-145-5p in tissue were determined by qRT-PCR. Cells were transfected with control mimics and miR-145-5p mimics for 24 or 48 h, and the RNA (24 h) and protein (48 h) expressions were assessed by qRT-PCR and Immunoblot respectively. All results are representative of at least three independent experiments and each value is the mean ± SD of three determinations; * *p* <  0.05, **** *p*  <  0.001.

To investigate the mechanism of CP upregulation in lung cancer, small RNAs were sequenced from seven specimens taken from lung cancer patients and miRNA-CP interactions were predicted using the miRwalk 2.0 website. The result revealed that miR-145-5p could bind with a sequence in the CP 3’untranslated region (UTR) (Figure 4A). Seven-paired samples were analyzed for miR-145-5p expression, and lower levels of miR-145-5p expression were observed in 5 out of 7 tumor samples compared with the normal lung samples, as determined by RNA-seq (Figure 4B). The GSE63805 dataset, which consisted of 30 non-tumor adjacent tissues and 32 lung adenocarcinoma tissues, also reveals lower levels of miR-145-5p expression in the lung adenocarcinoma samples (Figure 4C). The levels of miR-145-5p expression were different in the CL1-0, CL1-5, H1299, H1435 and H1563 cell lines and negatively correlated with CP expression in five lung cancer cell lines (Figure 2A and Figure 4D), with an R^2^ value of 0.6854 (*p* = 0.0419) (Figure 4D,E). These results indicate that the expression of CP was under the epigenetic regulation of miR-145-5p.

Next, we confirmed miR-145-5p-CP interaction using miRNA mimics and inhibitors transfection. CP expression decreased in both mRNA and protein levels, while H1563 cells were transfected with miR-145-5p mimics (5 and 10 nM) (Figure 4F,G). Overexpression of miR-145-5p also decreased CP expression in human embryonic kidney (HEK)-293 cells (Figure 4H). In contrast, miR-145-5p inhibitors enhanced CP expression in both H1299 and CL1-5 cell lines, which had a higher endogenous miR-145-5p and lower endogenous CP levels (Figure 2A and Figure 4I). Direct binding between miR-145-5p and CP was also confirmed by reporter analysis using miR-145-5p overexpressing HEK-293 cells co-transfected with miR-145-5p and either CP 3’UTR or mutant (MT) 3’UTR-luciferase reporter plasmid. Luciferase activity notably decreased following miR-145-5p overexpression, compared with control miRNA cDNA plasmid transfection (Figure 4J). These findings indicate that miR-145-5p inhibits CP expression in lung cancer via a direct interaction. 

### 2.5. miR-145-5p Inhibited HIF-2α Expression Leads to the Suppression of Angiogenesis

**Figure 5:**
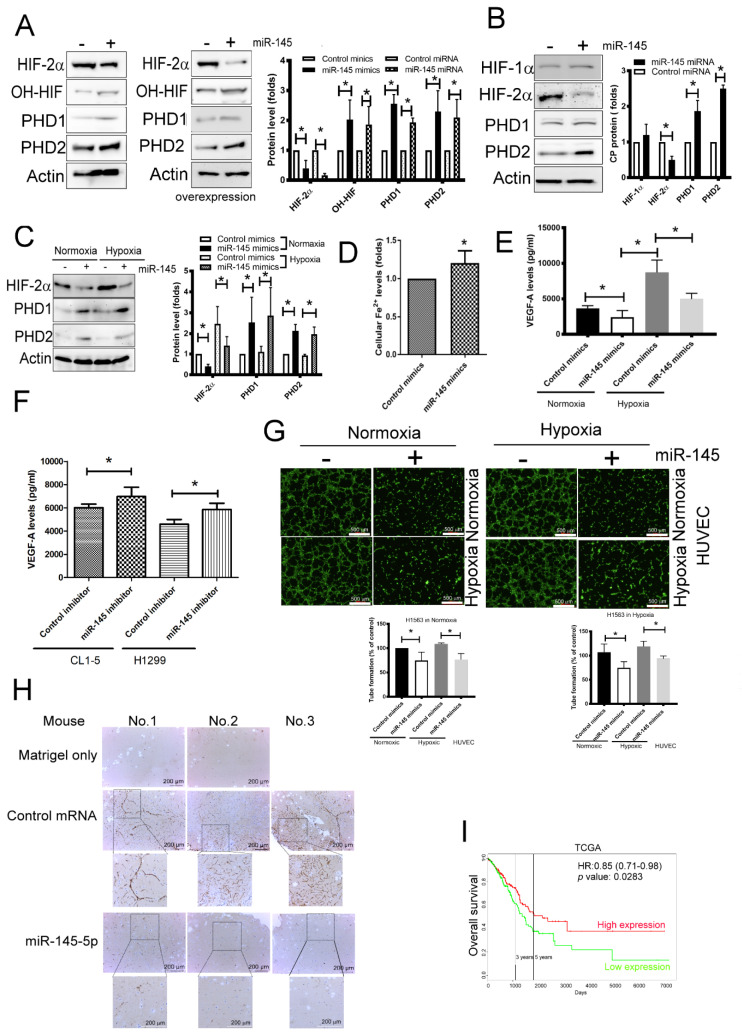
miR-145-5p decreased tumor angiogenesis by regulating CP-mediated signaling. (**A**,**B**) The effect of miR-145-5p in HIF-2α and PHD’s expression in H1563 and HEK-293 cells. (**C**) miR-145 mimics decreased the level of HIF-2α in H1563 cell in either a normoxic or hypoxic condition. (**D**,**E**) miR-145-5p increased cytosolic Fe^2+^ levels and decreased VEGF-A expression. (**F**) miR-145-5p inhibitors increased VEGF-A expression in CL1-5 and H1299 cells. (**G**,**H**) Elevated miR-145-5p reduced H1563-derived tumor angiogenesis in vitro and in vivo. (**I**) The relationship of miR-145-5p and the clinical outcome of lung cancer patients. Lung cancer cells were transfected with control mimics or miR-145-5p mimics, control inhibitors or miR-145-5p inhibitors for 48 h, and the protein expressions were assessed by Immunoblot. Alternatively, miR-145-5p overexpressing H1563 cells were established by cDNA transfection and G418 selection. The conditioned media of control mimics and miR-145-5p mimics that transfected H1563 cells were collected after 48 h incubation. HUVECs were seeded into Matrigel-coated well containing various conditioned media (50%). The tube formation was visualized by Calcein-AM dye. The level of VEGF-A was determined by Luminex Assays. Either of control or miR-145-5p overexpressing H1563 cells were mixed with high concentration of Matrigel and then subcutaneously injected into nude mice (Martrigel control *n* = 2, others *n* = 6). After 28 days, the Matrigel plugs were collected and dissected, then analyzed by IHC using CD34 antibody. All results are representative of at least three independent experiments and each value is the mean ± SD of three determinations; * *p*  <  0.05.

As miR-145-5p regulated CP expression, it was explored whether miR-145-5p contributes to the metabolism of HIF-2α by changing iron/PHD coupling. As shown in Figure 5A, both transient miR-145-5p mimics transfection and stable miR-145 overexpression increased the levels of PHD1/2 and HIF hydroxylation following the decrease of HIF-2α level. Similarly, overexpression of miR-145-5p also decreased the level of HIF-2α by increasing PHD1/2 expression and HIF hydroxylation in HEK-293 cells (Figure 5B). In addition, the regulation of miR-145-5p mimics in the enhancement of PHD1/2 and their function was also observed in H1563 cells under either a normoxic or hypoxic condition (Figure 5C). Consistent with the results observed after CP knockdown in H1563 cells, miR-145-5p mimics transfected increased ferrous (Fe^2+^) levels in H1563 (Figure 5D). In addition, the levels of VEGF-A expression declined in H1563 cells after transfection with miR-145-5p mimics in both normoxic and hypoxic conditions, however, IL-8, PDGF-AA, Ang-1 and angiogenin were not significantly altered (Table 2 and Figure 5E). In contrast, transfection of miR-145-5p inhibitors enhanced VEGF-A expression in both CL1-5 and H1299 lung cancer cells (Figure 5F). Transfection of miR-145-5p mimics decreased tube formation of HUVECs induced by H1563-CM collected under either a normoxic or hypoxic condition (Figure 5G). Furthermore, Matrigel plug angiogenesis assay showed that angiogenesis was attenuated while H1563 cells expressed high-level miR-145-5p, compared with the wild-type H1563 in a mouse model (Figure 5H). Survival analysis performed using PROGMmiRV2, a miRNA prognostics database, revealed an increased survival time in groups with high levels of miR-145-5p expression compared with groups with low levels of miR-145-5p expression (Figure 5I). These observations indicate that miR-145-5p decreases the levels of HIF-2α by increasing PHD activity, which in turn inhibits tumor angiogenesis in lung cancer.

## 3. Discussion

Growing evidence indicates that tumor angiogenesis is not only a consequence of cancer growth, but a contributor to its entire development from initial cancer growth at the primary site to terminal stage cancer metastasis as well [23,24,25]. As HIFs are critical regulators in the induction of tumor angiogenesis [26], an investigation of how HIFs are expressed (as cancer-promoters) in cancer cells under either a normoxic or hypoxic condition is warranted. The current study provides evidence that the upregulation of CP caused by a loss of miR-145-5p reduces HIF-2α degradation and leads to lung cancer angiogenesis. The regulatory loop of miR-145-5p and CP confirms that miR-145-5p and CP might be predictive biomarkers or promising strategies for lung cancer treatment. 

CP mediates several physiological functions, such as copper transport, iron oxidation, etc. [16] Serum CP levels have been linked to lung cancer occurrence. In addition, levels of CP in tumor were correlated with lung cancer invasiveness and prognosis [27]. High CP expression was also found in other cancer cells, such as breast and colon carcinoma [20]. However, the molecular mechanism by which CP contributes to lung cancer progression has not been well investigated. Higher levels of CP mRNA expression were detected in the 4 out of 7-paired tumor/normal lung tissues and the 7 datasets from the Oncomine^®^ database. More importantly, higher levels of CP were significantly correlated with a shorter relapse free survival and overall survival in lung cancer patients from the KM-Plotter and GEPIA databases. This demonstrated that high CP expression could be positively associated with death related events and shows that the CP protein acts as a potential oncogenic factor, leading to lung cancer progression and poor clinical outcomes.

CP, a ferroxidase [16], expresses as two splice variants, including a secreted (sCp) plasma enzyme and a membrane-bound glycosylphosphatidylinositol-anchored (GPI-Cp) protein [28]. Both of sCP and GPI-CP have been report to regulate iron efflux via oxidation [29]. Ferrous ion (Fe^2+^) is essential for regulating enzymatic activity in DNA synthesis, DNA repair and epigenetic regulation, as well as the control of cell proliferation; however it also enhances tumor growth [30]. Fe^2+^ is incorporated into PHD as a co-factor to hydroxylate HIF-α, and this iron controls the HIF and WNT signaling pathways in cancer [31,32,33]. In this study, the PHDs expression increased after CP knockdown. The CP affected both Fe^2+^ and PHDs amount. HIF-2α has been previously reported to be linked with various disease processes in cancer development, including tumorigenesis, proliferation, metastasis, tumor angiogenesis and chemoresistance [33,34,35]. Enhancement of HIF-2α has been linked to cancers in brain, head/neck, melanoma, lung and confers poor prognosis [10]. A recent study indicates that deficiency of CP decreases iron efflux, resulting in iron accumulation in astrocytes [36]. Our data indicated both sCP and GPI-CP are overexpressed in lung cancer, although the sCP level was 4-fold higher than GPI-CP. The results of this current study indicated that knockdown of CP, both sCP and GPI-CP increases cellular Fe^2+^ levels and enhances PHD1/2, which in turn causes the hydroxylation of HIFs. PHD inhibitor DMOG and proteasome inhibitors MG-132 prevented the effects of CP knockdown in HIF-2α stabilization, thereby indicating that CP modulates HIF-2α levels in an iron/PHD cascade-dependent degradation. CP could regulate HIF-2α levels in either a normoxic or hypoxic condition, indicating elevated CP may contribute HIF-2α over-activation in an oxygen-independent events. This provides the pathogenic explanation that oncogenic role of HIFs in cancers locate in non-hypoxic microenvironment. In summary, the results of the present study reveal a novel molecular mechanism that CP upregulates HIF-2α via the interruption of PHD/iron coupling in both normoxic and hypoxic conditions.

Knockdown of CP in H1563 cells did not significantly affect their proliferation, migration or invasion abilities. Unexpectedly, tumor stem cells did not show a significant difference after CP knockdown, although HIF-2α has been reported to increase the property of cancer stem cells in lung cancer [37]. In the current study, the most critical role of HIF-2α in lung cancer cells was to promote angiogenesis, which enhanced malignant cells to adapt to hypoxic microenvironments [38]. Knockdown of CP attenuated angiogenesis, as supported by the tube formation of HUVECs in H1563-conditioned medium and an in-vivo Matrigel plug angiogenesis assay. The molecular characterization of angiogenic growth factors, such as VEGF-A, IL-8, PDGF-AA, Ang-1 and angiogenin, which are driven by HIF-2α, have been identified as key factors of cancer-related angiogenesis [39]. However, only VEGF-A decreased in cell supernatants following CP knockdown; IL-8, PDGF-AA, Ang-1 and angiogenin were not significantly different. These results suggest that CP mediated angiogenesis occurs via VEGF-A in lung cancer. 

miRNAs control all parts of cancer biology, including cell proliferation, apoptosis, invasion, metastasis and angiogenesis [40,41]. Loss of function in tumor suppressive miRNAs might cause oncogene overexpression, resulting in cancer development [42]. A previous study suggested that miR-145-5p might be a tumor suppressor via regulation of angiopoetin-2 [40]. Our results and GEO dataset showed that lower miR-145-5p expression in the tumor of patients with lung adenocarcinoma. In addition, the direct regulatory loop of miR-145-5p-CP protein was supported by several evidences, including negative correlation between miR-145-5p and CP, the binding capacity of miR-145-5p on CP 3’-UTR, and the inhibitory effect of ectopic miR-145-5p in CP expression. Consistently, a restoring role of miR-145-5p was found in all consequences caused by CP dysregulation, including imbalance of Fe^2+^, PHD1/2 inactivation, then increased HIF-2α levels in lung cancer cells, even in normoxic microenvironments. Consequently, tumor angiogenesis was attenuated by miR-145-5p due to its inhibition of the HIF-2α/VEGF-A axis. Furthermore, lung cancer patients with higher levels of miR-145-5p survive longer. The current study demonstrates that a loss of miR-145-5p is critical for the pro-tumor function of CP, by increasing tumor angiogenesis in lung cancer.

## 4. Materials and Methods

### 4.1. Cell Lines

*Human normal lung cells HBE135-E6E7 (HEB),* human adenocarcinoma cell lines (H1435, H1563 and H1299), human embryonic kidney HEK293 cells, human bronchial epithelial Beas-2B cells and human umbilical vein endothelial cells (HUVECs) were obtained from American Type Culture Collection (ATCC, Manassas, VA, USA). CL1-0 and CL1-5 cells were kindly provided by Dr. Pan-Chyr Yang of National Taiwan University. H1437, H1563, H1299, CL1-0 and CL1-5 cells were cultured in RPMI1640 medium (Lonza, Basel, Switzerland) supplemented with 10% fetal bovine serum (FBS) (Thermo Fisher Scientific, Boston, MA, USA ), 100 U/mL penicillin and 100 μg/mL streptomycin (Thermo Fisher Scientific, Boston, MA, USA). Beas-2B cells were culture in BEBM medium (ATCC, Manassas, VA, USA). HEK293 cells were cultured in Eagle’s Minimum Essential Medium with 10% FBS, 100 U/mL penicillin and 100 μg/mL streptomycin (Thermo Fisher Scientific, Boston, MA, USA). HUVECs were cultured in complete vascular cell basal medium supplemented with an endothelial cell growth kit (ATCC, Manassas, VA, USA). For the hypoxic experiments, H1563 cells were cultured in either a normoxic (21% oxygen) or hypoxic (1% oxygen) condition for 24 h. The pharmacological PHD inhibitor dimethyloxallyl glycine (DMOG, Sigma-Aldrich, St. Louis, MO, USA) and proteasome inhibitor MG-132 (Sigma-Aldrich, St. Louis, MO, USA) were used at a working concentration of 75 and 10 μM, respectively. All cells were authenticated by short tandem repeat (Promega, Madison, WI, USA) and examined for mycoplasma contamination using a MycoAlert™ mycoplasma detection kit (Lonza, Basel, Switzerland) every three months.

### 4.2. CP Knockdown, miRNA Mimics and Inhibitors Transfection and miR-145-5p Overexpression

Knockdown of CP inH1563 cells was performed using a lentiviral expression system obtained from the National RNAi Core Facility (Taipei, Taiwan). Overexpression of miR-145-5p in H1563 and HEK-293 cells was achieved by transfecting the miR-145-5p DNA plasmid (GeneCopoeia, Rockville, MD, USA) using Lipofectamine 2000 reagent (Thermo Fisher Scientific, Boston, MA, USA). Stable clones of cells with CP knockdown and miR-145-5p overexpression were established by puromycin selection. The cells were transfected with control mimics, miR-145-5p mimics (5 or 10 nM), control inhibitors or miR-145-5p inhibitors (10 nM) using DharmaFECT 1 Transfection Reagent (Dharmacon, Lafayette, CO, USA) or *Lipofectamine RNAiMAX* (Thermo Fisher Scientific, Boston, MA, USA). Oligonucleotides with random sequences served as negative controls for the miRNA mimics and inhibitors (Dharmacon, Lafayette, CO, USA). 

### 4.3. Analysis of Proangiogenic Factors

The supernatants of H1563, H1299 and CL1-5 cells transfected with shRNA plasmids, miRNA mimics or inhibitors were harvested (24 h incubation) and cell debris was removed by centrifugation for 10 min at 3000 rpm for further analysis. The levels of various proangiogenic factors were measured using Luminex Assays (R&D Systems, Minneapolis, MN, USA). 

### 4.4. RNA-Sequencing and Quantitative Real-Time Polymerase Chain Reaction (qRT-PCR) and Patient Data

Seven pairs of adjacent non-tumor lung and tumor tissues were obtained from the Division of Thoracic Surgery and Division of Pulmonary and Critical Care Medicine, Kaohsiung Medical University Hospital (Kaohsiung, Taiwan). This study protocol was reviewed and approved by the Institutional Review Board of Kaohsiung Medical University Hospital (KMUH-IRB-20130054, 24 May 2013). The mRNA and microRNA profiles were examined using next-generation sequencing (NGS) (Welgene Biotechnology Company, Taipei, Taiwan) [43]. Total RNA was isolated from cells using TRIzol Reagent (Life Technologies, Boston, MA, USA). miRNAs and cDNAs were reverse transcribed using Mir-X™ miRNA First Strand Synthesis (Clontec, Mountain View, CA, USA) and reverse transcriptase (Takara, Shiga, Japan) kits, respectively. RNA levels were determined using real-time analysis with SYBR Green on a StepOne-Plus machine (Applied Biosystems, Foster City, CA, USA). The relative expression levels of specific mRNA and miRNA were normalized to glyceraldehyde 3-phosphate dehydrogenase or U6 snRNA, respectively. The relative standard method (2^−ΔΔ*C*t^) was used to calculate relative RNA expressions. The following primers were used: CP (forward, 5′-TGGAAAATCCCAGAAAGATCTG-3′ and reverse, 5′-AGGGCAAATTCCAGTTTCCT-3′); sCP (forward, 5′-5′TCCCTGGAACATACCAAACC3′-3′ and reverse, 5′-CCAATTTATTTCATTCAGCCGA-3′); GPI-CP (forward, 5′-′ACCCCGAGAAAGTAAACAAAGATG′-3′ and reverse, 5′-GATTGGGTAGATCACATTCCATA-3′) [44]; miRNA-145-5p (GTCCAGTTTTCCCAGGAATCCCT); and glyceraldehyde 3-phosphate dehydrogenase (GAPDH) (forward, 5′-TTCACCACCATGGAGAAGGC-3′ and reverse, 5′-GGCATGGACTGTGGTCATGA-3′).

Gene expression data and corresponding clinical information data of NSCLC patients were downloaded from the publicly available database Gene Expression Omnibus (GEO, http://www.ncbi.nlm.nih.gov/geo/) for validation of CP expression. The GSE31210 consisted of a total of 226 lung adenocarcinoma cases. These 226 primary lung adenocarcinoma at stage I-II consisted of *EGFR, KRAS, ALK* mutations and triple negative [45,46]. The GSE63805 dataset consisted of 30 non-tumor adjacent tissues and 32 lung adenocarcinoma tissues without mentioning mutation status [47].

### 4.5. Analysis of CP 3′UTR Activity

HEK-293 cells overexpressing miR-145-5p were transfected with the CP reporter plasmid containing the predicted miR-145-5p binding sites (Origene, Rockville, MD, USA) or mutated miR-145-5p binding sites/Renilla luciferase plasmid (Promega, Madison, WI, USA) (10:1) using Lipofectamine 2000 (Thermo Fisher Scientific, Boston, MA, USA) according to the manufacturer’s protocol. The cells were washed, and luciferase activity was determined using a Dual-Luciferase Reporter Assay System (Promega, Madison, WI, USA). Relative luciferase activity was first normalized by Renilla activity, and then compared with those of the respective controls.

### 4.6. Immunoblot

Cellular total protein was extracted using RIPA lysis buffer (EMD Millipore, Billerica, MA, USA) supplemented with a protease inhibitor cocktail (Sigma-Aldrich, St. Louis, MO, USA). An equal amount of cellular protein was denatured by heating, and then separated by SDS-PAGE. Proteins were transferred to PVDF membranes (EMD Millipore, Burlington, MA, USA) and probed with various primary antibodies for 4-16 h, followed by incubation with horseradish peroxidase (HRP)-conjugated secondary antibodies (Cell-Signaling Technology, Danvers, MA, USA). Signals of specific proteins were detected using a chemiluminescence kit (EMD Millipore, Burlington, MA, USA). The primary antibodies used included those against CP (ab48614, Abcam, Cambridge, UK), PHD2, PHD3 (Catalog #ab30782) and PHD1 (Catalog #ab113077), and they were obtained from Abcam (Cambridge, UK). Anti-HIF-2α (catalog no. 4691), and -PHD2 (Catalog #4835) antibodies were purchased from Cell-Signaling Technology (Danvers, MA, USA). HIF-1α (Catalog #610959) antibody was purchased from BD Biosciences (San Jose, CA, USA). Actin (catalog no. MABT825) and GAPDH (catalog no. MAB374) antibodies were obtained from EMD Millipore (Burlington, MA, USA). Hydroxyl-HIF pro402) (catalog no.07-1585) was obtained from Sigma-Aldrich (St. Louis, MO, USA). The quantitation result of the Immunoblot was performed using AlphaImager software (Alpha Innotech, San Leandro, CA, USA). Full gel was shown in Figures S2–S4.

### 4.7. Cell Proliferation, Transwell Migration, Wound Healing and Tumor Spheroid Formation

Cell proliferation was assessed by WST-1 and BrdU incorporation (EMD Millipore, Burlington, MA, USA) analysis, according to the manufacturer’s protocol. For migration, cells were seeded into inserts with polyester membranes with a pore size of 8 μm (EMD Millipore, Burlington, MA, USA). Complete cell culture medium was then added to the bottom wells for 48 h as a chemo-attractant. Migratory cells were visualized using crystal violet staining. Alternatively, cells were placed in to a 12 well-pate at 100% confluence, and the cell movement was measured by determine the migration of cells into the acellular area formatted by a sterile tip. The cells were stained with PKH26 dye and then seeded in ultralow-attachment plates (Corning Life Sciences, Tewksbury, MA, USA) for tumor sphere formation. After 14 days, the tumor spheres were assessed using an inverted Nikon Eclipse TE300 microscope (Nikon, Minato, Tokyo Japapn). The quantitation results of transwell migration and spheroid formation were performed using counting method.

### 4.8. Tube Formation Analysis of Endothelial Cells

HUVECs (4 × 10^4^ cells/well) were seeded onto growth factor-reduced BD Matrigel (1 mg/mL) (BD Biosciences, San Jose, CA) in a 48-well plate. The HUVECs were supplemented with the supernatants (50%) of the lung cancer cells transfected with CP shRNA or miR-145-5p mimics/inhibitors. The HUVECs were then stained with *Calcen AMi dye* (Thermo Fisher Scientific, Boston, MA, USA) for 30 min and imaged using an inverted Nikon Eclipse TE300 microscope (Nikon, Minato, Tokyo Japapn).  The quantitation result of tube formation was performed using Image J.

### 4.9. In Vivo Matrigel Plug Assay and Immunohistochemistry (IHC)

H1563 cells (1 × 10^6^ cells/well) which stably expressed CP shRNA or miR-145-5p were mixed with ice-cold high concentration Matrigel (400 µL, BD Biosciences, San Jose, CA). This solution was then subcutaneously injected into nude mice (male, 8-week-old BALB/c, *n* = 6, Matrigel control, *n* = 2). After 28 days, the Matrigel plugs were collected and dissected, then analyzed using IHC with CD34 rabbit polyclonal antibodies (Abcam, Cambridge, UK). All animal care was in accordance with institutional guidelines (Approval number: 105114, 15 December 2016). 

### 4.10. Statistical Analysis

Results are presented as mean ± standard deviation (SD). Multiple group comparisons were performed using two-way analysis of variance (ANOVA) with Tukey’s post-hoc test. The analyses were performed using GraphPad Prism (GraphPad Software Version 7, San Diego, CA). Two tested groups were compared using a Student’s *t*-test. A *p*-value < 0.05 was considered to be statistically significant.

## 5. Conclusions

**Figure 6:**
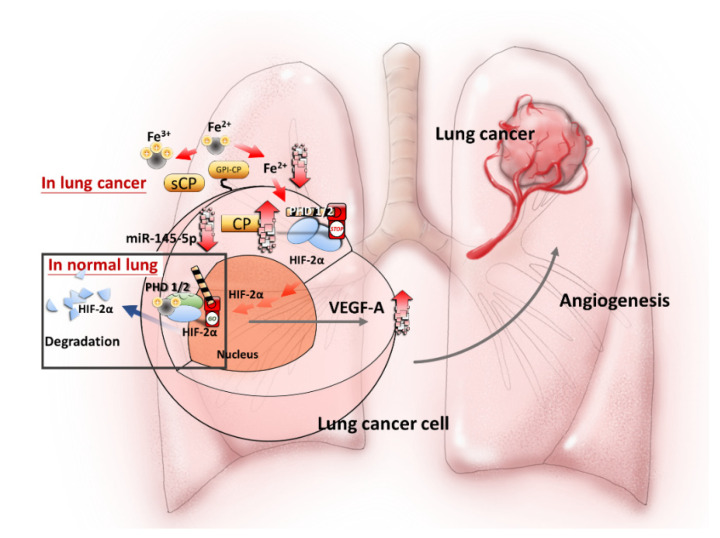
Proposed model of miR-145-5p-CP dysregulation in lung cancer. In normal lung, lower CP (secreted CP (sCP) and glycosylphosphatidylinositol-anchored CP (GPI-CP)) expression leads to ferrous ion (Fe^2+^) accumulation, PHD 1/2 activation and followed by HIF-2α degradation. However, in lung cancer, lower miR-145-5p preserves CP, which oxidizes Fe^2+^ into Fe^3+^ with the consequent inactivation of PHD1/2. The inactivation of PHDs in turn increases HIF-2α stabilization. The elevated HIF-2α increases VEGF-A expression, resulting in tumor angiogenesis. Our study provides a new mechanism by which CP dysregulation due to loss of function in miR-145-5p is involved in the angiogenesis in lung cancer.

Taken together, the results of the current study reveal that CP elevation contributes to a decrease of Fe^2+^ and followed by HIF-2α activation via PHDs and angiogenesis, which in turn supports cancer growth and metastasis. The results identify a new mechanism by which cancer cells enhance angiogenesis via a previously unexplored miR-145-5p→CP→PHD1/2→HIF-2α→VEGF-A signaling pathway (Figure 6). This process of epigenetic dysregulation, which includes oncogenic HIF-derived proangiogenic factors in malignant cells and the endothelium, might provide novel targets for preventative therapies and may allow for the development of personalized medicine for lung cancer patients.

## Figures and Tables

**Table 1:** The level of various pro-angiogenic factors in H1563 and CP-knockdown H1563 cells.

Cell Line: H1563	CP Protein	
Angiogenic Factor (pg/mL)	Control shRNA	CP shRNA
IL-8	16,595.8 ± 487.2	15,364.1 ± 415.6
PDGF-AA	1036.8 ± 897.4	1049.9 ± 525.8
Ang-1	1262.7 ± 194.9	1232.5 ± 172.3
Angiogenin	1762.7 ± 1031.2	1529.7 ± 885.7

**Table 2:** The effect of miR-145-5p in the expression of various pro-angiogenic factors in lung cancer.

Cell Line: H1563	miR-145-5p			
Angiogenic Factor (pg/mL)	Control Mimics		miR-145 Mimics	
IL-8	11,245.8 ± 897.4		12,832.8 ± 800.7	
PDGF-AA	1375.6 ± 971.4		1458.1 ± 339.8	
Ang-1	905.1 ± 297.3		829.2 ± 93.9	
Angiogenin	2042.8 ± 1058.7		1733.8 ± 490.9	
	**miR-145 Inhibitor**			
**Cell Lines**	**CL1-5**		**H1299**	
**Angiogenic Factor (pg/mL)**	**Control Inhibitor**	**miR-145 Inhibitor**	**Control Inhibitor**	**miR-145 Inhibitor**
IL-8	17,521 ± 839.8	17,300.8 ± 541.7	548.4 ± 251.6	562.4 ± 309.8
PDGF-AA	4223.7 ± 2323.1	4391.8 ± 2327.8	17 ± 3.1	17.3 ± 3.1
Ang-1	525.9 ± 163.9	471.192 ± 109.4	679.9 ± 197.6	625.6 ± 129.6
Angiogenin	10,980.4 ± 1014.1	10,280.5 ± 1917.3	2352.2 ± 951.2	2409.0 ± 1226.4

## Supplementary Materials

Supplementary materials can be found at https://www.mdpi.com/1422-0067/21/14/5081/s1.

## Abbreviations

CP	Ceruloplasmin
HIF	Hypoxia-inducible factor
*PHD*	*Prolyl hydroxylase*
NSCLC	Non-small cell lung cancer
EGFR	Epidermal growth factor receptor
ALK	Anaplastic lymphoma kinase
DMOG	Dimethyloxalylglycine
HUVECs	Human umbilical vein endothelial cells
PDGF-AA	Platelet-derived growth factor-AA
*VEGF*	*Vascular endothelial growth factor*
UTR	Untranslated region
