# Supplementary material for: Loss of miR-145-5p Causes Ceruloplasmin Interference with PHD-Iron Axis and HIF-2α Stabilization in Lung Adenocarcinoma-Mediated Angiogenesis
Source: Int J Mol Sci. 2020 Jul 18;21(14):5081. doi: 10.3390/ijms21145081 (PMC7404111; doi:10.3390/ijms21145081)
Supplement: Supplementary file 1 [file ijms-21-05081-s001.pdf]

Supplementary Files:

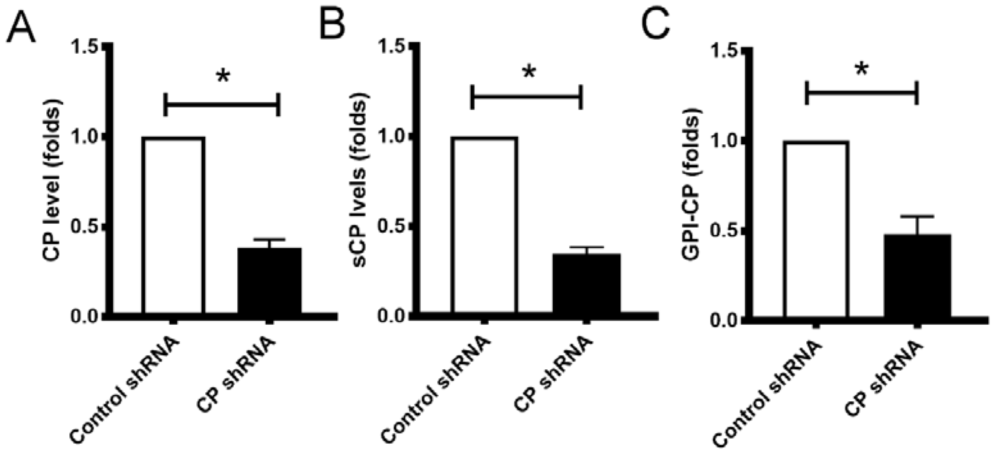

sFigure 1. The knockdown efficacy of CP shRNA in H1563 cells. The levels of (A) total CP, (B) sCP and (C) GPI-CP mRNA in H1563 cells. The results are representative of at least three independent experiments and each value is the mean ± SD of three determinations; **p* < 0.05.

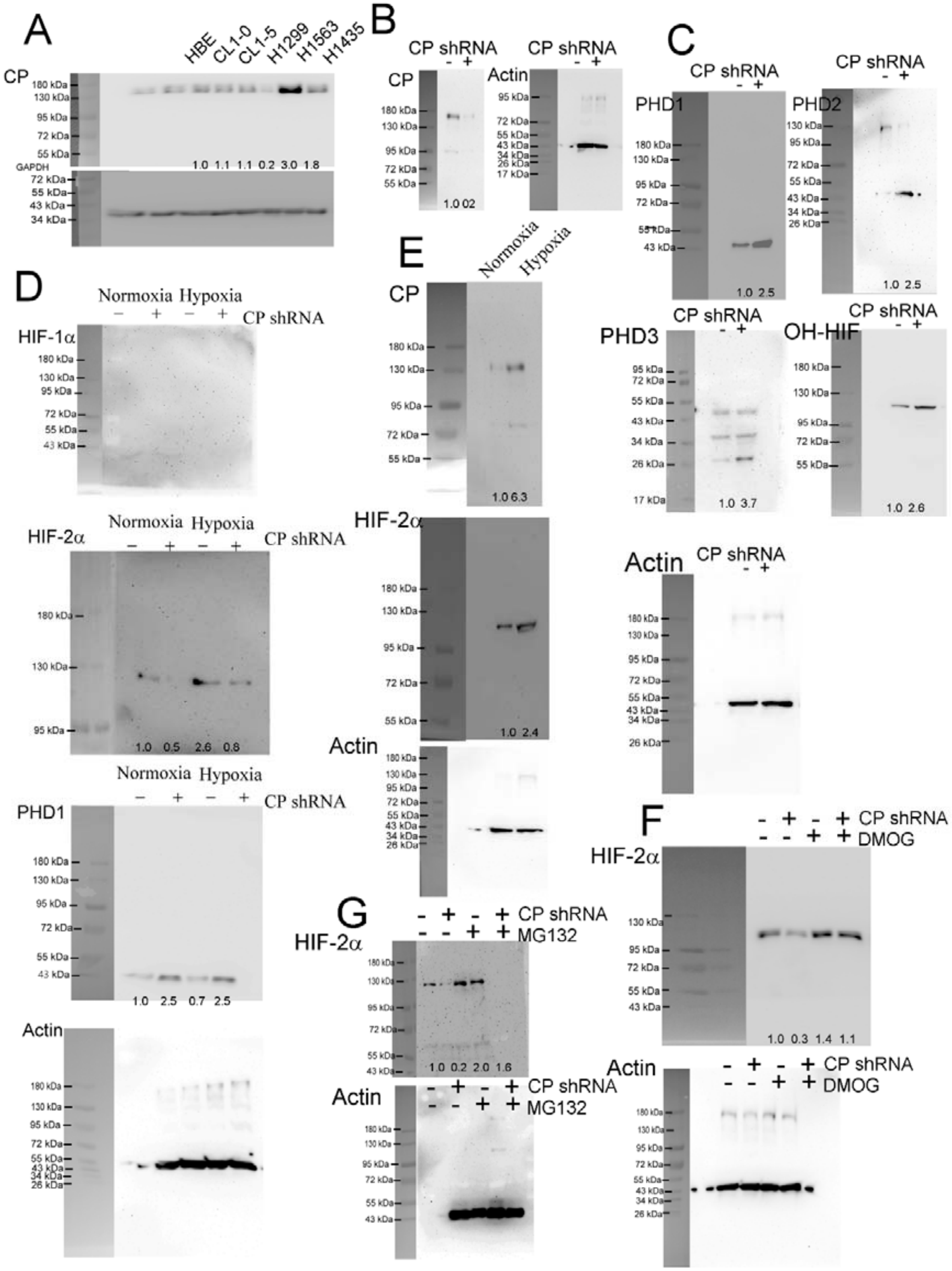

sFigure 2. The expression of CP and HIFs in normal and cancer cell lines. (A) The levels of CP at mRNA in different cell lines. (B) The expression of CP after CP shRNA plasmid transfection. (C) The levels of HIFs and PHDs in CP knockdown H1563 cells. (D) CP affected HIF-2α stabilization in either a normoxic or hypoxic condition. (E) Hypoxia induced CP expression. (F) A PHD inhibitor and (G) a proteasome inhibitor reversed the effect of CP knockdown in HIF-2α degradation.

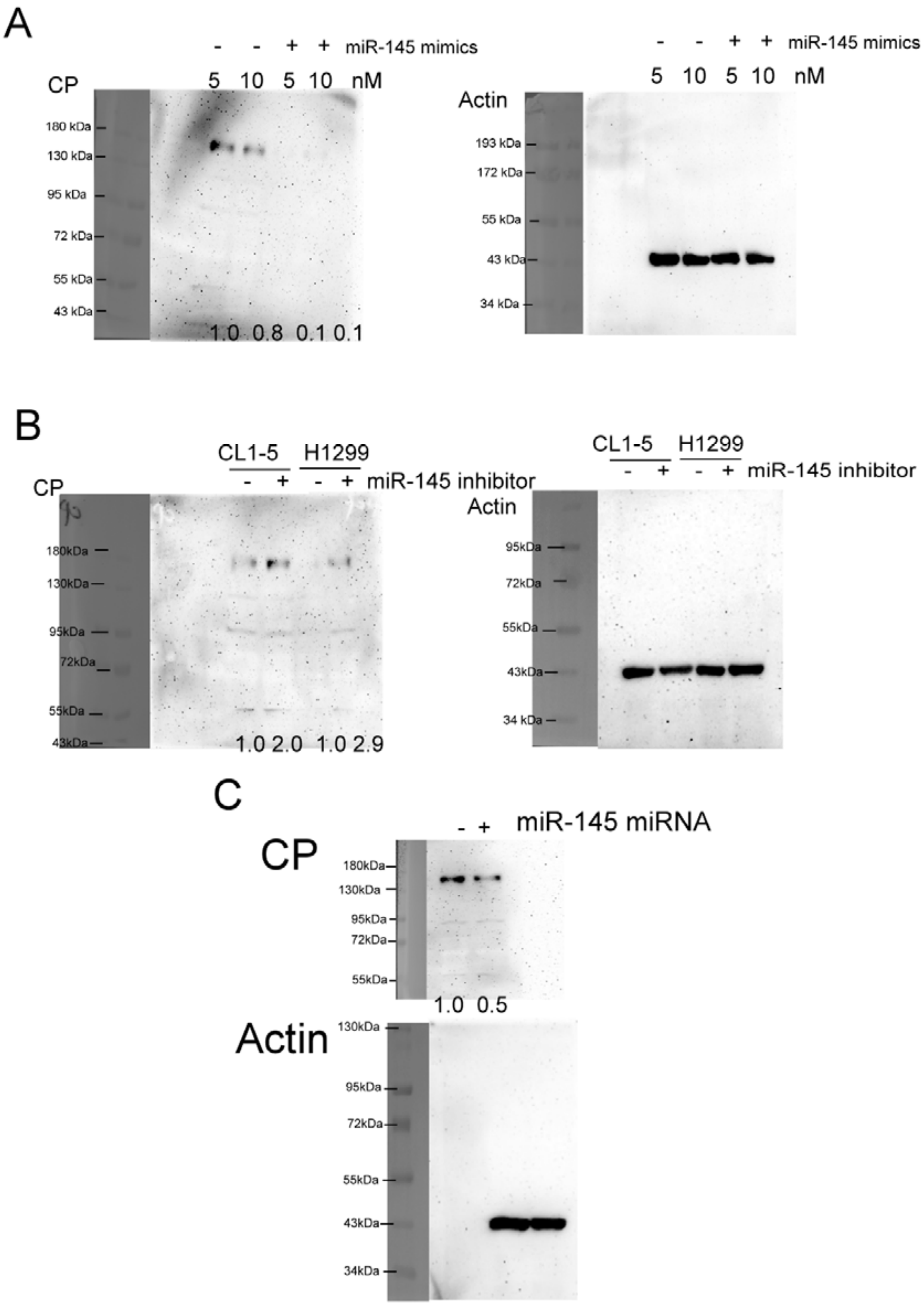

sFigure 3. CP is the target of miR-145-5p. (A) miR-145-5p mimics reduced CP protein expression in H1563 cells. (B) miR-145-5p inhibitors enhanced CP protein expression in H1299 and CL1-5 lung cancer cell lines. (C) Ectopic expression of miR-145-5p reduced the expression of CP in HEK-293 cells. The levels of specific proteins were detected by Immunoblot.

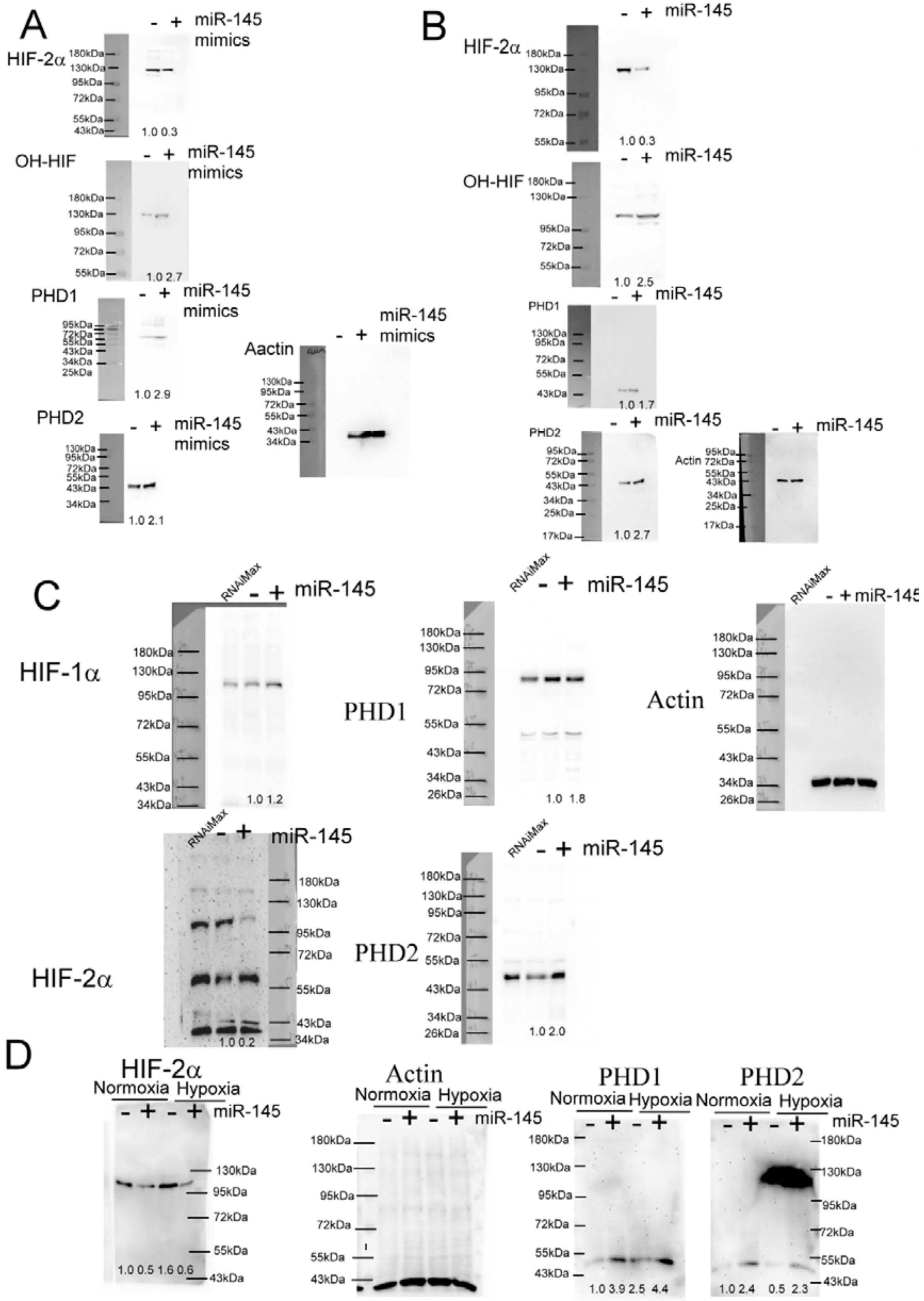

sFigure 4. miR-145-5p regulated CP-mediated signaling. The effect of miR-145-5p on HIF-2α and PHDs expression in H1563 by (A) mimics transfection and (B) stable overexpression. (C) Transfection of miR-145-cDNA in HEK-293 cell changed the level of HIFs. (D) CP regulated HIF-2α in either a normoxic or hypoxic condition. The levels of specific proteins were detected by Immunoblot.
